# Complete Genome Sequences of Two Porcine Deltacoronavirus Strains from Henan Province, China

**DOI:** 10.1128/MRA.01517-18

**Published:** 2019-03-07

**Authors:** Qingqing Liang, Bingxiao Li, Honglei Zhang, Hui Hu

**Affiliations:** a College of Animal Science and Veterinary Medicine, Henan Agricultural University, Zhengzhou, Henan, People’s Republic of China; b Key Laboratory for Animal-Derived Food Safety of Henan Province, Zhengzhou, Henan, People’s Republic of China; University of Southern California

## Abstract

In 2016 and 2018, two porcine deltacoronavirus (PDCoV) strains, CH-01 and HNZK-02, were identified from fecal samples of piglets with diarrhea in Henan Province, China. The full-length genomic sequence analysis indicated that these two strains had high nucleotide identities with the other Chinese PDCoV epidemic strains.

## ANNOUNCEMENT

Porcine deltacoronavirus (PDCoV) is an enveloped, single-stranded positive-sense RNA virus, belonging to the genus *Deltacoronavirus* in the family *Coronaviridae* of the order *Nidovirales* (1). PDCoV has the smallest genome in the known coronaviruses and is approximately 25.4 kb in size (1). PDCoV mainly causes acute diarrhea, vomiting, dehydration, and mortality in neonatal piglets. This virus was first discovered in swine rectal swabs in Hong Kong in 2012 (2). In 2014, a PDCoV outbreak in swine herds was first reported in Ohio (3, 4). So far, PDCoV has been reported in the United States, China, South Korea, Laos, Vietnam, Thailand, Canada, and Japan (5 – 7).

In our study, we collected several fecal samples from piglets that had clinical signs of diarrhea in Hebi and Zhoukou regions in Henan Province, China, and two PDCoV strains, CH-01 and HNZK-02, were identified from these samples in February 2016 and March 2018, respectively. The swine feces were 5-fold diluted with phosphate-buffered saline (PBS), vortexed, and centrifuged at 1,847 × *g* for 30 min at 4°C. Total RNA was extracted from the supernatant samples using TRIzol reagent (Invitrogen, Carlsbad, CA, USA) according to the manufacturer’s instructions, and PDCoV was tested by reverse-transcription PCR (RT-PCR) using primers targeting the PDCoV N gene. Thirteen pairs of primers were designed to amplify the full-length genome of PDCoV based on the HKU15-155 strain (GenBank accession number JQ065043) (Table 1). The number of amplified DNA fragments was 1 amplicon per primer set. The total viral RNA was reverse transcribed with oligo(dT) primer. The 13 overlapping amplicons spanning the entire PDCoV genome were amplified using the PrimeSTAR GXL DNA polymerase (TaKaRa). Using PDCoV-23804-F and oligo(dT) as the rapid amplification of cDNA ends (RACE) primers, the 3′ terminal sequence was amplified by PCR. The amplified fragments were sequenced twice by the Sanger method on an ABI 3730xl DNA analyzer (Applied Biosystems). The complete sequences were obtained by assembling overlapping contigs, followed by trimming off primer sequences using the EditSeq and MegAlign programs of DNAStar 7.0 green (DNAStar, Madison, WI). The 13 amplicons of the sequenced PDCoVs were aligned to the reference strain HKU15-44 using the Clustal W program of DNAStar 7.0 green, and open reading frames (ORFs) were determined by sequence comparison. The range of lengths of the 13 amplicons are shown in Table 1.

**TABLE 1:** Primers used for amplification of the complete genome

Primer name^*a*^	Sequence (5′–3′)	Amplified length (bp)	Nucleotide position range	*T_m_*^*b*^ (°C)
PDCoV-1-F	ACATGGGGACTAAAGATAAAAATTATAGC	3,300	1–3300	58
PDCoV-3300-R	GCTCATCGCCTACATCAGTG	3,300	1–3300	58
PDCoV-3091-F	CGGATTTAAAACCACAGACT	1,770	3091–4860	51
PDCoV-4860-R	ACGACTTTACGAGGATGAAT	1,770	3091–4860	51
PDCoV-4741-F	CTCCTGTACAGGCCTTACAA	1,680	4741–6420	55
PDCoV-6420-R	TCACACGTATAGCCTGCTGA	1,680	4741–6420	55
PDCoV-6291-F	CTCAATGCAGAAGACCAGTC	1,751	6291–8041	53
PDCoV-8041-R	CAGCTTGGTCTTAAGACTCT	1,751	6291–8041	53
PDCoV-7920-F	GGTACTGCTTCTGATAAGGAT	1,741	7920–9660	53
PDCoV-9660-R	TAGGTACAGTTGTGAACCGA	1,741	7920–9660	53
PDCoV-9463-F	TACTCTTCACAGCCTTCAC	1,668	9463–11130	51
PDCoV-11130-R	GCAAATCCAGGACCCATAGTAG	1,668	9463–11130	51
PDCoV-10560-F	CGCTACACTATTGTGAAGAA	2,288	10560–12847	52
PDCoV-12847-R	TTCGTAGGGCTCAAGATA	2,288	10560–12847	52
PDCoV-12301-F	TCCAGATGACCGTTGCGTAT	2,652	12301–14952	55
PDCoV-14952-R	CCAACAGAGTCGGGTAAA	2,652	12301–14952	55
PDCoV-14703-F	CACCCATAACGAAGAACC	2,483	14703–17185	54
PDCoV-17185-R	CCGATGAGTGTCGTAGCG	2,483	14703–17185	54
PDCoV-17165-F	TGCCGCTACGACACTCAT	2,039	17165–19203	56
PDCoV-19203R	TCCGCTAAAGGAGAATAGGTTG	2,039	17165–19203	56
PDCoV-18485-F	TGCTACCCAATCTTACAGT	2,595	18485–21079	53
PDCoV-21079-R	GCAAATACTCCGTCTGAAC	2,595	18485–21079	53
PDCoV-20761-F	GTCTTACCGTGTGAAACCCC	3,065	20761–23825	55
PDCoV-23825-R	AACCACGAGACTGTAAGCAA	3,065	20761–23825	55
PDCoV-23804-F	TTTTGCTTACAGTCTCGTGGTT	1,616	23804–25419	55
Oligo(dT)	TTTTTTTTTTTTTTTTTT	1,616	23804–25419	55

a Each primer was designed based on the HKU15-155 strain (GenBank accession number JQ065043).

b *T_m_*, melting temperature.

The genomic sequence of the PDCoV strain CH-01 was identified as having 25,404 nucleotides (nt), and the PDCoV strain HNZK-02 was identified as being 25,419 nt in length, excluding the 3-poly(A) tail. The genomic G+C contents of the two sequenced PDCoV strains (CH-01 and HNZK-02) were 43.18% and 43.06%, respectively. The genomic structure of the two PDCoVs contained the following gene order: 5′ untranslated region (UTR), open reading frame 1a/1b (ORF 1a/1b), spike glycoprotein (S), envelope (E), membrane (M), nonstructural protein 6 (Nsp6), nucleocapsid (N), Nsp7, and 3′ UTR. The sizes of these genes of the PDCoV strain CH-01 were 539, 18,788, 3,480, 252, 654, 285, 1,029, 603, and 392 nt, respectively. The PDCoV strain HNZK-02 contained an 18,803-nt ORF1a/1b, which had a 6-nt and 9-nt insert at nucleotide positions 1739 and 2804, respectively, compared with that of the CH-01 strain (the nucleotide positions were numbered according to the entire genome sequence of PDCoV CH-01). The other genes in strain HNZK-02 had the same sizes as those of the PDCoV CH-01 strain.

The identities of PDCoV nucleotide and deduced amino acid sequences were aligned and analyzed using the Clustal W program of DNAStar 7.0 green. The two sequenced PDCoV strains have nucleotide identities from 98.1% to 99.4% with the Chinese epidemic PDCoV strains (GenBank accession numbers KP757892, KR131621, MF280390, KU981062, and KP757890) and nucleotide identities from 98.9% to 99.0% with some U.S. PDCoV strains (GenBank accession numbers KJ481931, KJ462462, KJ567050, and KJ769231).

The sequence data of the two PDCoV strains will facilitate future research on the epidemiology and evolutionary biology of PDCoV in China.

### Data availability.

The complete genome sequences of the two PDCoV strains (HNZK-02 and CH-01) have been deposited in GenBank under the accession numbers MH708123 and KX443143.
